# Impact of individual and neighborhood social capital on the physical and mental health of pregnant women: the Japan Environment and Children’s Study (JECS)

**DOI:** 10.1186/s12884-020-03131-3

**Published:** 2020-08-06

**Authors:** Ryoko Morozumi, Kenta Matsumura, Kei Hamazaki, Akiko Tsuchida, Ayako Takamori, Hidekuni Inadera, the Japan Environment and Children’s Study (JECS) Group, Michihiro Kamijima, Shin Yamazaki, Yukihiro Ohya, Reiko Kishi, Nobuo Yaegashi, Koichi Hashimoto, Chisato Mori, Shuichi Ito, Zentaro Yamagata, Hidekuni Inadera, Takeo Nakayama, Hiroyasu Iso, Masayuki Shima, Youichi Kurozawa, Narufumi Suganuma, Koichi Kusuhara, Takahiko Katoh

**Affiliations:** 1 Faculty of Social Sciences, University of Toyama, 3190 Gofuku, Toyama-shi, Toyama, 930-8555 Japan; 2 Toyama Regional Center for JECS, University of Toyama, Toyama, Japan; 3 Department of Public Health, Faculty of Medicine, University of Toyama, Toyama, Japan

**Keywords:** Social capital, SF-8, Inverse probability weighting, Average treatment effect, Pregnant women, Japan Environment and Children’s Study

## Abstract

**Background:**

Previous studies revealed positive, negative, and no influence of social capital on the health outcomes of pregnant women. It was considered that such differences were caused by the disparities of outcome measures and sample sizes between studies. Our chief aim was to verify the positive influence of social capital on the health condition of pregnant women using established health outcome measures and large-scale nationwide survey data.

**Methods:**

We employed questionnaire survey data from 79,210 respondents to the Japan Environment and Children’s Study, and physical and mental component summary scores from the 8-Item Short-Form Health Survey as outcome measures. We estimated the effect of individual and neighborhood social capitals on physical and mental component summary scores. To consider the property that the richness of social capital would be generally determined by individual characteristics, and to estimate the causal influence of social capital on health without bias caused by said property, we adopted average treatment effect estimation with inverse probability weighting. Generally, average treatment effects are based on the difference of average outcomes between treated and untreated groups in an intervention. In this research, we reckoned individuals’ different levels of social capital as a kind of non-randomized treatment for respective individuals, and we applied average treatment effect estimation. The analysis regarded pregnant women with the lowest level of social capital as untreated samples and women with other levels of social capitals as treated samples.

**Results:**

For mental component summary score, the maximum average treatment effects in the comparison between the lowest and highest levels of social capital were approximately 4.4 and 1.6 for individual and neighborhood social capital, respectively. The average treatment effects for the physical component summary score were negligible for both social capital types.

**Conclusions:**

Social capital particularly contributes to improving mental component summary score in pregnant women. The likelihood of a mentally healthy pregnancy may be increased by enhancing social capital.

## Background

The influence of social capital on health has been widely discussed in existing literature; however, these previous studies have produced conflicting findings regarding whether social capital (in the form of social networking and cohesion) can, through communication and social support, enhance health outcomes [1].

We analyzed the effects of social capital on women’s health during pregnancy. In Japan, the social environment surrounding pregnant women has evolved in recent decades, with existing trends toward nuclear families, rural depopulation, and higher urban population densities being considered to have weakened intergenerational exchanges and community ties [2]. Importantly, it is unclear whether such weakened social ties negatively affect health during pregnancy. In particular, if there is a negative association between a lack of access to social capital and healthy pregnancy, this could mean that current societal trends are having a significant negative overall effect in this regard. Conversely, if social capital has a positive impact on health during pregnancy, it could, in conjunction with standard medical interventions, offer a means of alleviating physical and mental difficulties for pregnant women.

Previous studies have investigated the effect of social capital on several indicators of health during pregnancy, including self-rated health [3]; 12-Item Short-Form Health Survey (SF-12) scores [4]; symptoms of depression and anxiety [4–9]; pregnancy complications [4, 10, 11]; oral-health-related quality of life (OHRQoL) [12]; preterm birth [4, 8, 11, 13–22]; and low birth weight [4, 13, 20, 21]. Of these investigations, those of self-rated health, SF-12 scores, symptoms of depression and anxiety, pregnancy complications, and OHRQoL have reported that social capital has a favorable positive influence in this regard. However, the analyses of the influence of social capital on preterm birth and low birth weight have produced inconsistent results, with some studies reporting positive relationships [4, 8, 11, 20–22] and others reporting no effect or a negative effect [13–19].

In the most relevant study to the present research, the SF-12 was administered to pregnant women in Berlin, and social support was consequently found to have a positive influence on their scores [4] (the SF-12 is a shorter version of the 36-Item Short-Form Health Survey [SF-36], which is widely used to measure functional health and well-being, and is based on self-reports from respondents). However, this previous study was conducted across a limited geographic area and examined fewer than 1000 respondents; thus, analysis of large-scale nationwide data is needed to assess the generalizability of the researchers’ findings.

Considering this, we sought to clarify the impact of social capital on health during pregnancy using a large nationwide sample. More specifically, our study analyzed data gathered by the Japan Environment and Children’s Study (JECS), which is a nationwide birth cohort study sponsored by the Ministry of the Environment of Japan. The primary aim of the JECS is to analyze the effect of environmental risk factors on children’s health, and the project is being conducted in 15 regional centers across Japan (Hokkaido, Miyagi, Fukushima, Chiba, Kanagawa, Koshin, Toyama, Aichi, Kyoto, Osaka, Hyogo, Tottori, Kochi, Fukuoka, and south Kyushu/Okinawa). As part of JECS, researchers recruited expectant mothers from these areas between 2011 and 2014, of which approximately 100,000 pregnancies registered. The project will continue to follow these parents and children until the children reach 13 years of age. The design of the JECS has been reported in detail elsewhere [23–25].

We used nationwide survey data from the JECS to investigate the impact individual and neighborhood social capital have on the physical and mental component summary (PCS and MCS, respectively) scores of the 8-Item Short-Form Health Survey (SF-8; another short version of the SF-36). This research is expected to make two main contributions: first, the results should clarify whether social capital has a positive impact on health; and second, the results obtained from this large-scale dataset should reveal general attributes of the Japanese population. Previous studies of social capital have discussed both its positive and negative effects. Examples of negative impacts would be the exclusion of outsiders and strong enforcement of local norms [1, 26, 27]. To determine its true impact, the effect of social capital should be verified based on validated measurement scores and data. If the scale of the positive impact is smaller than that of the negative impact, this may indicate that social capital has an overall negative impact. Conversely, if our investigation finds that the positive impact is large and statistically significant, this could contribute to the promotion of health-care policies that focus on the social capital of pregnant women.

## Methods

### Study design

The JECS gathered medical records, questionnaire results, and biological specimens from pregnant women from pregnancy through to child-rearing, with the content of the data collected depending on the stage of gestation, parturition, and childcare. This method of investigation enabled researchers to determine participants’ characteristics throughout the period in question.

We used the data obtained from the questionnaires and medical records. Pregnant women completed the first questionnaire (M-T1) during their first trimester, and the second questionnaire (M-T2) during their second and third trimesters. These respondents answered the questionnaires and returned them in person at subsequent prenatal visits or by sending them via mail to JECS Regional Centers. Where possible, the centers addressed incomplete questionnaires by performing subsequent face-to-face or telephone interviews with the respondents [24]. The participating women also recruited their partners, and there are approximately half as many registered fathers in the dataset as there are registered mothers. We limited the data used in our analysis to the mothers’ responses; this was to avoid the risk of sample selection bias that could be caused by including fathers’ responses. M-T1 includes question items concerning family characteristics, disease, tobacco use, substance use, working status, working environment, and various other topics. Moreover, M-T2 contains question items pertaining to health status, dietary habits, tobacco use, sleep quality, home appliances, substance use, working status, education history, household income, and social capital. Finally, medical records following delivery (Dr-0 m) contain details regarding the newborn baby, obstetric and delivery complications, and other topics.

From M-T1, we used the information regarding family characteristics, self-reported history of disease, and labor-force participation; moreover, from M-T2 we used the PCS and MCS scores, age, experience of stressful events, education history, household income, and level of social capital (stressful events included experiencing, over the course of the previous year, the death and/or illness of a loved one, the loss of the respondent’s and/or spouse’s job, the acquiring of a significant mortgage, divorce, moving home, and marital problems). The presence of obstetric complications was identified using information from M-T2 and Dr-0 m. In Dr-0 m, physicians reported the timing and diagnosis of obstetric complications; if the diagnosis of an obstetric complication was recorded prior to the respondent completing M-T2, we regarded the respondent as having experienced a pregnancy with an obstetric complication.

### Outcome measures

In our statistical analysis, we considered the PCS and MCS scores as outcome variables. Specifically, the SF-8 PCS and MCS scores were calculated based on the respondents’ answers to the question items in M-T2, which includes items assessing general health, physical functioning, role-physical, bodily pain, vitality, social functioning, mental health, and role-emotional. The PCS and MCS scores measure physical and mental functioning, respectively, with higher scores indicating better health status; the validity of the Japanese translation of these question items has been verified in previous research [28].

### Exposure

The main exposures are the variables measuring pregnant women’s social capital. As previous studies conceptualized, we regarded the resources embodied by the individual’s social network as individual social capital; and the resources formed by social cohesion, such as the stocks of trust or reciprocal relationships within the community, as neighborhood social capital [3, 12, 29]. The M-T2 questionnaire contained question items pertaining to individual communication and evaluation of trust in and support received from neighbors.

Supplementary Table 1 (Additional File 1) shows the question items related to social capital. The contents of the questions concerning individual social capital (questions A to D) are similar to those of the six questions from the Social Support Questionnaire (SSQ) [30]. In response to these questions, the respondents provide information regarding how often and strongly they depend on others. The variables we extracted from the questions represented social capital in terms of social networking at the individual level. Moreover, the questions on neighborhood social capital (questions E and F) are similar to the “social cohesion and trust” components of a questionnaire used in the Project on Human Development in Chicago Neighborhoods (PHDCN) [31]. Questions E and F require respondents to evaluate their degree of trust in and the support they receive from their neighbors. The answers respondents provide to these questions imply group attributes, measured in terms of individual understanding. The variables we extracted from these questions were considered to reflect social cohesion

### Participants

**Fig. 1:**
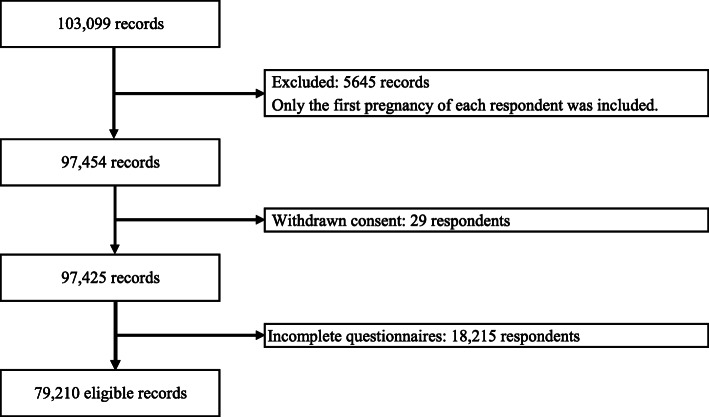
Study flow chart

Research groups can access the JECS data through the JECS Program Office’s regional centers. We used the “jecs-ag-20160424” dataset, which includes questionnaire responses from mothers and fathers and medical records from physicians from the time of registration to 1 month after parturition. Before beginning the statistical analysis, we excluded some portions of the dataset, in accordance with our research criteria. More specifically, the total number of pregnancies registered in the dataset was 103,099. Women registered with multiple pregnancies within the survey period were included but, for each woman, we limited the data to that for the first pregnancy, which reduced the dataset to 97,454. Data from respondents who withdrew consent were eliminated; this resulted in a further reduction to 97,425 participants. Finally, we targeted data from only participating women who answered all question items related to the variables used in our analysis. Thus, we ultimately analyzed a dataset of responses obtained from 79,210 respondents. The study flow chart is shown in Fig. 1.

### Statistical analysis

To estimate the causal influence of social capital on health, we adopted average treatment effect (ATE) estimation with an inverse probability weighting (IPW) estimator. In the analysis, it is required to consider the property that the richness of social capital would be endogenously determined by individual characteristics in general, and to measure the causal influence without bias caused by said property. Generally, ATE is based on the difference of average outcomes between treated and untreated groups in an intervention. In this research, we reckoned individuals’ different levels of social capital as a kind of non-randomized treatment for respective individuals, and we applied the method of the ATE estimation.

The IPW estimator is useful to control the bias caused by non-randomized treatment. In cases where the treatment is not randomized, a simple comparison of outcome averages between treated and untreated groups highlights both the effects of the treatment and the differences in characteristics between them. For the correction of the sample selection bias caused by the characteristic differences, the IPW estimator is valuable in ATE estimation.

We regarded pregnant women with the lowest level of social capital as untreated samples and the women with other levels of social capitals as treated samples in the ATE estimation with the IPW estimator. When the level of social capital is determined by individual characteristics, the characteristics would differ between treated and untreated samples. Our analysis used the IPW estimator to avoid the bias caused by the characteristic differences.

In IPW, the reciprocal of the probabilities of the assignment to the treated and untreated groups for respective samples are estimated and employed as the weighting variables in the calculation of averages within the groups. The variables control the effect of the non-randomized assignment on the averages.

The IPW estimator requires a regression equation for assigning samples between the treated and untreated groups. As a dependent variable, the equation has a dichotomous variable that is equal to “1” for treated samples and “0” for untreated samples. The individual characteristics considered to affect the sample assignment are used as the independent variables. The results of this estimation provide for each individual predicted probabilities regarding their level of belonging to the treated and untreated groups, respectively; this predicted probability is generally called a “propensity score.” The inverse probability, namely, the reciprocal of the propensity score, is used in the IPW estimator. Thus, we respectively calculated the weighted averages for the treated and untreated samples using the inverse probabilities as the weighting variables.

The mathematical specification of the IPW estimator is as follows. For individual *i*, *z*_*i*_ is a dichotomous variable that reflects his/her assignment to the treated and untreated groups, respectively. If a sample is assigned to the treated group, *z*_*i*_ = 1; if the sample is assigned to the untreated group, *z*_*i*_ = 0. ***x***_***i***_ represents the vector of the covariates for the assignment. The predicted probability that the sample is assigned to the treated group is described as *e*_*i*_ = *p*(*z*_*i*_ = 1| ***x***_***i***_), and the range of the probability is 0 to 1. This probability is individual *i*‘s propensity score. The predicted probability that the sample is assigned to the untreated group is 1 − *e*_*i*_. These predicted probabilities are generally obtained from the results of logistic regression analysis. When *y*_*i*_ is an outcome of individual *i*, the weighted average of the outcome variable among the treated samples using the inverse probability is $\hat{E}\left({y}_1\right)=\sum \limits_{i=1}^{N_1}\frac{z_i{y}_i}{e_i}/\sum \limits_{i=1}^{N_1}\frac{z_i}{e_i}$. Moreover, the weighted average among the untreated samples is $\hat{E}\left({y}_0\right)=\sum \limits_{i=1}^{N_0}\frac{\left(1-{z}_i\right){y}_i}{1-{e}_i}/\sum \limits_{i=1}^{N_0}\frac{\left(1-{z}_i\right)}{1-{e}_i}$. The ATE is calculated by $\hat{E}\left({y}_1\right)-\hat{E}\left({y}_0\right)$ [32–37].

In the survey from which our data are sourced, the question items pertaining to social capital present three or more response choices, allowing for different levels of social capital. Based on their answers to these questions, the samples are divided into groups representing various levels of social capital. Each answer is transformed into a categorical variable. We calculated the predicted probability through multinomial logistic regression, with the categorical variable set as a dependent variable. The group with the lowest level of social capital was regarded as the untreated group; the other groups were regarded as the treated groups. The analysis considered the ATE between the groups with the lowest and some different medium levels of social capital, as well as the ATE between the groups with the lowest and highest levels of social capital.

The following example illustrates the method we used to calculate the ATE, based on a question item for which an individual selects an answer from three options. Specifically, one of the items on the questionnaire regarding social capital is: “The number of friends or neighbors to whom you can talk casually about your concerns” (Supplementary Table 1, Additional File 1). The associated response options are: “none,” “one or two,” and “three or more.” This question item creates three groups with different levels of social capital. In our analysis process, first, the multinomial logistic regression analysis is performed, which provides the probabilities that the respective options are selected by an individual. When the predicted probabilities are described as *e*_*i*0_ (for “none”), *e*_*i*1_ (for “one or two”), and *e*_*i*2_ (for “three or more”), respectively, for individual *i*, *e*_*i*0_ + *e*_*i*1_ + *e*_*i*2_ = 1. We calculate the weighted averages of the outcome variable for the respective groups using the reciprocal of the predicted probabilities. The weighted average of the outcome among untreated samples who select “none” is: $\hat{E}\left({y}_0\right)=\sum \limits_{i=1}^{N_0}\frac{y_i}{e_{i0}}/\sum \limits_{i=1}^{N_0}\frac{1}{e_{i0}}$. Similarly, the weighted averages among treated samples who select “one or two” and “three or more” are expressed as: $\hat{E}\left({y}_1\right)=\sum \limits_{i=1}^{N_1}\frac{y_i}{e_{i1}}/\sum \limits_{i=1}^{N_1}\frac{1}{e_{i1}}$ and $\hat{E}\left({y}_2\right)=\sum \limits_{i=1}^{N_2}\frac{y_i}{e_{i2}}/\sum \limits_{i=1}^{N_2}\frac{1}{e_{i2}}$, respectively. The ATEs are calculated by $\hat{E}\left({y}_1\right)-\hat{E}\left({y}_0\right)$ and $\hat{E}\left({y}_2\right)-\hat{E}\left({y}_0\right)$.

To calculate ATE based on the IPW estimator, two assumptions must be satisfied, otherwise this ATE evaluation cannot be justified [38]. One assumption is that each sample has a positive possibility of receiving each treatment level. When there is at least some overlap between the estimated density of the propensity scores that treated samples are assigned to an untreated group and the estimated density of the propensity scores that untreated samples are assigned to an untreated group, the overlap assumption is not violated. If the estimated density for the treated samples has most of its mass near 0, while for the untreated samples the estimated density is near 1, these densities do not have an overlapping region, and the overlap assumption is violated [38, 39].

The second assumption is that the means of the covariates corrected by the IPW estimator are balanced between treated and untreated samples. When the means of the covariates of the treated samples are close to those of the untreated samples, the assumption can be considered as not being violated [38, 40]. Before obtaining the ATEs, the validity of these assumptions must be checked.

All analyses were performed using the STATA MP software package, version 15.0 (STATA Corporation, College Station, TX).

## Results

The summary statistics of the variables we used are shown in Table 1. These summary statistics indicate the individual characteristics of the participating pregnant women. The mean PCS and MCS scores were 45.64 and 49.13, respectively. Most participants were married (including common-law marriage). Approximately half did not have children prior to the current pregnancy. Of the pregnant women in this sample, over 80% had been diagnosed with at least one type of disease, approximately 20% had experienced at least one obstetric complication, and over 40% reported experiencing at least one stressful event in the past year.

**Table 1:** Background characteristics of the participants

Characteristics	Respondents	n (%)
The 8-item Short-Form Health Survey		
PCS scores: mean (range)	45.64	(5.72–65.45)
MCS scores: mean (range)	49.13	(12.87–73.33)
Age (years): mean (range)	31.19	(15–47)
Marital status		
Unmarried, divorced, or bereaved	2896	(3.66)
Married or in a common-law relationship	76,314	(96.34)
Number of children		
0	36,813	(46.48)
≥ 1	42,397	(53.52)
Self-reported history of disease		
No	12,416	(15.67
Yes	66,794	(84.33)
Obstetric complications		
No	64,957	(82.01)
Yes	14,253	(17.99)
Experience of any stressful events		
No	44,535	(56.22)
Yes	34,675	(43.78)
Labor force participation		
No	25,481	(32.17)
Yes	53,729	(67.83)
Mother’s academic history		
Junior high or high school	27,181	(34.32)
Technical college or vocational school	19,461	(24.57)
College, university, or graduate school	32,568	(41.12)
Father’s academic history		
Junior high or high school	33,870	(42.76)
Technical college or vocational school	16,219	(20.48)
College, university, or graduate school	29,121	(36.76)
Household income (million JPY/year)		
< 2	4200	(5.30)
2–4	27,004	(34.09)
4–6	26,364	(33.28)
6–8	12,819	(16.18)
8–10	5366	(6.77)
≥ 10	3457	(4.36)
Individual social capital		
A: Is there someone available to you who shows you love and affection?		
None of the time	3310	(4.18)
A little of the time	6127	(7.74)
Some of the time	22,244	(28.08)
Most of the time	6583	(8.31)
All of the time	40,946	(51.69)
B: Is there someone whom you can count on for emotional support (discuss prolems or help you make a difficult decision)?		
None of the time	1675	(2.11)
A little of the time	5859	(7.40)
Some of the time	15,852	(20.01)
Most of the time	8171	(10.32)
All of the time	47,653	(60.16)
C: How often do you have a desired level of contact with someone whom you feel close to, trust, and can confide in?		
None of the time	1198	(1.51)
A little of the time	10,316	(13.02)
Some of the time	30,605	(38.64)
Most of the time	14,794	(18.68)
All of the time	22,297	(28.15)
D: Number of friends or neighbors with whom you can casually share your concerns		
None	786	(0.99)
One or two	30,184	(38.11)
Three or more	48,240	(60.90)
Neighborhood social capital		
E: Neighbors trust each other.		
Disagree	16,107	(20.33)
Somewhat disagree	19,625	(24.78)
Somewhat agree	36,802	(46.46)
Agree	6676	(8.43)
F: Neighbors help each other.		
Disagree	15,687	(19.80)
Somewhat disagree	19,619	(24.77)
Somewhat agree	36,326	(45.86)
Agree	7578	(9.57)

*PCS* Physical Component Summary, *MCS* Mental Component Summary

The summary statistics also revealed the sample profiles of individual and neighborhood social capital. For questions A to F, responding “none of the time,” “none,” or “disagree” indicated the lowest level of social capital. First, regarding individual social capital (questions A to D), for questions A to C 10–15% of the pregnant women responded “none of the time” or “a little of the time,” thereby indicating that they had low levels of individual social capital. Moreover, the responses to question D indicated that approximately 40% of the respondents did not have three or more friends or neighbors with whom they could casually discuss their concerns. Regarding neighborhood social capital, responding “disagree” or “somewhat disagree” to questions E and F indicated low levels of neighborhood social capital; overall, approximately 45% of respondents answered “disagree” or “somewhat disagree” to these questions.

A two-sample t-test that compared the respective mean health statuses of the two groups showed that both PCS and MCS scores were related to age, marital status, disease, obstetric complications, stressful events, labor-force participation, mothers’ and fathers’ academic histories, and household income (Table 2). For individual and neighborhood social capital, the difference in the two-sample mean was generally greater for the MCS score than for the PCS score. In addition, the group with a higher level of social capital had better physical and mental health statuses, with a few exceptions.

**Table 2:** Comparison of means between groups of pregnant women (PCS and MCS scores)

Score type		PCS score			MCS score		
Characteristics	n	M	SD	*p*	M	SD	*p*
Age (years)							
< 35	58,144	45.89	6.12	< 0.001^a^	49.05	6.22	< 0.001^a^
≥ 35	21,066	44.94	6.44		49.34	6.20	
Marital status							
Unmarried, divorced, or bereaved	2896	46.48	6.28	< 0.001^a^	47.12	6.73	< 0.001^a^
Married or in a common-law relationship	76,314	45.61	6.22		49.21	6.19	
Number of children							
0	36,813	45.84	6.16	< 0.001^a^	49.17	6.27	0.073
≥ 1	42,397	45.46	6.26		49.09	6.18	
Self-reported history of disease							
No	12,416	46.72	5.89	< 0.001^a^	49.61	5.83	< 0.001^a^
Yes	66,794	45.44	6.26		49.04	6.28	
Obstetric complications							
No	64,957	46.02	5.92	< 0.001^a^	49.36	6.06	< 0.001^a^
Yes	14,253	43.88	7.20		48.09	6.78	
Experience of any stressful events							
No	44,535	45.87	6.04	< 0.001^a^	50.26	5.70	< 0.001^a^
Yes	34,675	45.34	6.43		47.68	6.55	
Labor force participation							
No	25,481	46.01	6.22	< 0.001^a^	49.20	6.15	0.037^b^
Yes	53,729	45.46	6.21		49.10	6.25	
Mother’s academic history							
Junior high or high school, technical college, or vocational school	46,642	45.77	6.20	< 0.001^a^	48.76	6.26	< 0.001^a^
College, university, or graduate school	32,568	45.46	6.24		49.66	6.11	
Father’s academic history							
Junior high or high school, technical college, or vocational school	50,089	45.75	6.21	< 0.001^a^	48.89	6.25	< 0.001^a^
College, university, or graduate school	29,121	45.45	6.24		49.55	6.13	
Household income (million JPY/year)							
< 4	31,204	46.10	6.09	< 0.001^a^	48.62	6.31	< 0.001^a^
≥ 4	48,006	45.34	6.29		49.46	6.14	
Individual social capital							
A: Is there someone available to you who shows you love and affection?							
None of the time, a little of the time	9437	45.81	6.25	0.004^a^	47.49	6.78	< 0.001^a^
Some of the time, most of the time, all of the time	69,773	45.61	6.22		49.35	6.11	
B: Is there someone whom you can count on for emotional support (discuss problems or help you make a difficult decision)?							
None of the time, a little of the time	7534	45.61	6.38	0.640	46.67	6.95	< 0.001^a^
Some of the time, most of the time, all of the time	71,676	45.64	6.20		49.39	6.08	
C: How often do you have a desired level of contact with someone whom you feel close to, trust, and can confide in?							
None of the time, a little of the time	11,514	45.64	6.26	0.937	47.51	6.72	< 0.001^a^
Some of the time, most of the time, all of the time	67,696	45.64	6.21		49.40	6.09	
D: Number of friends or neighbors with whom you can casually share your concerns							
None, one, or two	30,970	45.55	6.27	< 0.001^a^	48.15	6.47	< 0.001^a^
Three or more	48,240	45.70	6.19		49.76	5.97	
Neighborhood social capital							
E: Neighbors trust each other.							
Disagree, somewhat disagree	35,732	45.45	6.32	< 0.001^a^	48.51	6.48	< 0.001^a^
Somewhat agree, agree	43,478	45.79	6.13		49.64	5.94	
F: Neighbors help each other.							
Disagree, somewhat disagree	35,306	45.45	6.32	< 0.001^a^	48.60	6.48	< 0.001^a^
Somewhat agree, agree	43,904	45.79	6.14		49.56	5.97	

*PCS* Physical Component Summary, *MCS* Mental Component Summary

^a^*p* < 0.01, ^b^*p* < 0.05

The two-sample t-test of social capital revealed a correlation between social capital and certain individual characteristics (Table 3). More specifically, high individual social capital (measured in questions A to D) was found to be related to being married, having no previous children, experiencing no stressful events, having a higher education level, and having a higher household income (Table 3). Moreover, high neighborhood social capital (questions E and F) was found to be associated with older age, being married, having children, having no current disease, having no obstetric complications, experiencing no stressful events, not participating in the labor force, having a higher education level, and having a higher household income (Table 3).

**Table 3:** Comparison of means between groups of pregnant women

Individual social capital		A: Is there someone available to you who shows you love and affection?			B: Is there someone whom you can count on for emotional support (discuss problems or help you make a difficult decision)?		
Characteristics	n	M	SD	*p*	M	SD	*p*
Age (years)							
< 35	58,144	2.94	1.22	< 0.001^a^	3.20	1.12	< 0.001^a^
≥ 35	21,066	3.00	1.20		3.16	1.12	
Marital status							
Unmarried, divorced, or bereaved	2896	2.65	1.27	< 0.001^a^	2.91	1.22	< 0.001^a^
Married or in a common-law relationship	76,314	2.97	1.21		3.20	1.11	
Number of children							
0	36,813	3.01	1.19	< 0.001^a^	3.22	1.10	< 0.001^a^
≥ 1	42,397	2.91	1.24		3.16	1.13	
Self-reported history of disease							
No	12,416	2.86	1.25	< 0.001^a^	3.16	1.13	0.001^a^
Yes	66,794	2.97	1.21		3.20	1.12	
Obstetric complications							
No	64,957	2.96	1.22	0.041^b^	3.19	1.12	0.026^b^
Yes	14,253	2.94	1.21		3.17	1.12	
Experience of any stressful events							
No	44,535	2.99	1.21	< 0.001^a^	3.25	1.09	< 0.001^a^
Yes	34,675	2.92	1.22		3.11	1.15	
Labor force participation							
No	25,481	2.96	1.23	0.980	3.19	1.13	0.417
Yes	53,729	2.96	1.21		3.19	1.11	
Mother’s academic history							
Junior high or high school, technical college, or vocational school	46,642	2.82	1.25	< 0.001^a^	3.10	1.16	< 0.001^a^
College, university, or graduate school	32,568	3.15	1.14		3.32	1.05	
Father’s academic history							
Junior high or high school, technical college, or vocational school	50,089	2.87	1.24	< 0.001^a^	3.13	1.14	< 0.001^a^
College, university, or graduate school	29,121	3.11	1.16		3.30	1.07	
Household income (million JPY/year)							
< 4	31,204	2.79	1.26	< 0.001^a^	3.07	1.17	< 0.001^a^
≥ 4	48,006	3.06	1.18		3.27	1.07	
Response options for A and B: none of the time = 0, a little of the time = 1, some of the time = 2, most of the time = 3, all of the time = 4 ^a^*p* < 0.01, ^b^*p* < 0.05							
Individual social capital		C: How often do you have a desired level of contact with someone whom you feel close to, trust, and can confide in?			D: Number of friends or neighbors with whom you can casually share your concerns		
Characteristics	n	M	SD	*p*	M	SD	*p*
Age (years)							
< 35	58,144	2.60	1.08	< 0.001^a^	1.60	0.51	< 0.001^a^
≥ 35	21,066	2.55	1.07		1.58	0.52	
Marital status							
Unmarried, divorced, or bereaved	2896	2.47	1.11	< 0.001^a^	1.50	0.55	< 0.001^a^
Married or in a common-law relationship	76,314	2.59	1.07		1.60	0.51	
Number of children							
0	36,813	2.61	1.06	< 0.001^a^	1.61	0.51	< 0.001^a^
≥ 1	42,397	2.57	1.09		1.59	0.51	
Self-reported history of disease							
No	12,416	2.57	1.08	0.010^a^	1.60	0.51	0.888
Yes	66,794	2.59	1.07		1.60	0.51	
Obstetric complications							
No	64,957	2.59	1.08	0.768	1.60	0.51	0.270
Yes	14,253	2.59	1.07		1.59	0.51	
Experience of any stressful events							
No	44,535	2.64	1.07	< 0.001^a^	1.62	0.50	< 0.001^a^
Yes	34,675	2.52	1.08		1.57	0.52	
Labor force participation							
No	25,481	2.62	1.08	< 0.001^a^	1.59	0.52	< 0.001^a^
Yes	53,729	2.58	1.07		1.60	0.51	
Mother’s academic history							
Junior high or high school, technical college, or vocational school	46,642	2.50	1.08	< 0.001^a^	1.57	0.52	< 0.001^a^
College, university, or graduate school	32,568	2.72	1.05		1.64	0.49	
Father’s academic history							
Junior high or high school, technical college, or vocational school	50,089	2.53	1.08	< 0.001^a^	1.58	0.52	< 0.001^a^
College, university, or graduate school	29,121	2.69	1.06		1.62	0.50	
Household income (million JPY/year)							
< 4	31,204	2.50	1.09	< 0.001^a^	1.56	0.52	< 0.001^a^
≥ 4	48,006	2.65	1.06		1.63	0.50	
Response options for C: none of the time = 0, a little of the time = 1, some of the time = 2, most of the time = 3, all of the time = 4 Response options for D: none = 0, one or two = 1, three or more = 2 ^a^*p* < 0.01							
Neighborhood social capital		E: Neighbors trust each other.			F: Neighbors help each other.		
Characteristics	n	Mean	SD	*p*	Mean	SD	*p*
Age (years)							
< 35	58,144	1.38	0.91	< 0.001^a^	1.40	0.93	< 0.001^a^
≥ 35	21,066	1.57	0.87		1.58	0.86	
Marital status							
Unmarried, divorced, or bereaved	2896	1.16	0.94	< 0.001^a^	1.22	0.96	< 0.001^a^
Married or in a common-law relationship	76,314	1.44	0.90		1.46	0.91	
Number of children							
0	36,813	1.24	0.92	< 0.001^a^	1.25	0.93	< 0.001^a^
≥ 1	42,397	1.60	0.86		1.63	0.86	
Self-reported history of disease							
No	12,416	1.45	0.92	0.018^b^	1.48	0.93	< 0.001^a^
Yes	66,794	1.43	0.90		1.45	0.91	
Obstetric complications							
No	64,957	1.44	0.90	< 0.001^a^	1.46	0.91	< 0.001^a^
Yes	14,253	1.40	0.91		1.42	0.92	
Experience of any stressful events							
No	44,535	1.47	0.90	< 0.001^a^	1.48	0.91	< 0.001^a^
Yes	34,675	1.38	0.91		1.41	0.92	
Labor force participation							
No	25,481	1.54	0.89	< 0.001^a^	1.56	0.90	< 0.001^a^
Yes	53,729	1.38	0.91		1.40	0.92	
Mother’s academic history							
Junior high or high school, technical college, or vocational school	46,642	1.37	0.92	< 0.001^a^	1.41	0.93	< 0.001^a^
College, university, or graduate school	32,568	1.51	0.88		1.51	0.89	
Father’s academic history							
Junior high or high school, technical college, or vocational school	50,089	1.40	0.91	< 0.001^a^	1.44	0.92	< 0.001^a^
College, university, or graduate school	29,121	1.48	0.90		1.47	0.90	
Household income (million JPY/year)							
< 4	31,204	1.36	0.92	< 0.001^a^	1.40	0.93	< 0.001^a^
≥ 4	48,006	1.47	0.89		1.48	0.90	
Response options for E and F: disagree = 0, somewhat disagree = 1, somewhat agree = 2, agree = 3 ^a^*p* < 0.01, ^b^*p* < 0.05							

The ATEs for the PCS and MCS scores showed the degree that social capital affected physical and mental health. The weighted averages and ATEs were obtained through IPW estimation. The ATEs were obtained via subtracting the weighted average of the group with the lowest level of social capital from that of the group with other levels of social capital. The ATEs indicate how social capital influences the summary scores of the treated groups compared to the untreated group. If the ATE is positive and the magnitude is large, it can be concluded that social capital largely improves the summary score.

Before assessing the ATE values, we needed to verify the validities of the assumptions. We first found that the estimated densities of the propensity scores for the treated and untreated groups overlapped in the respective question items; this finding indicated that the overlap assumption was not violated. Second, we calculated the standardized differences, shown in Supplementary Tables 2-1 (Additional file 2), 2–2 (Additional file 3), and 2–3 (Additional file 4), as performed in previous research [38, 40]. These tables indicate the standardized difference between the means of the treated and untreated groups before and after correction by the IPW estimator. If the standardized difference calculated from the data weighted by the inverse probability is close to zero when compared with that from the raw data, the correction made to balance the treated and untreated groups can be regarded as appropriate. These tables show that the differences from the weighted data are approximately zero. Overall, the differences from the weighted data are smaller than those from the raw data; thus, we can conclude that the assumption of the balance between the treated and untreated groups was satisfied.

The ATEs for the PCS scores indicated a significant negative impact of social capital in question A, and a positive impact of social capital in question D (Table 4). Moreover, a positive impact of social capital on the PCS score was observed for neighborhood social capital (Table 4). For example, the ATEs for question E were 0.50 and 0.90 for “somewhat agree vs. disagree” and “agree vs. disagree,” respectively (a value of 0.90 indicates that the PCS score for respondents who most strongly feel mutual trust is 0.90 higher than that of respondents who feel no mutual trust). These tables show that the absolute values of the impacts of individual and neighborhood social capital on the PCS scores were between 0 and 1. These results indicate that social capital has a negligible effect on physical health during pregnancy.

**Table 4:** ATEs for PCS scores across different indicators of social capital

Individual social capital		COEF	SE	95% CI		*p*
A: Is there someone available to you who shows you love and affection?						
Weighted average	None of the time	46.10	0.11	45.87	46.32	< 0.001^a^
Weighted average	A little of the time	45.39	0.09	45.22	45.57	< 0.001^a^
Weighted average	Some of the time	45.55	0.04	45.47	45.63	< 0.001^a^
Weighted average	Most of the time	45.49	0.07	45.35	45.64	< 0.001^a^
Weighted average	All of the time	45.70	0.03	45.64	45.76	< 0.001^a^
ATE	A little of the time vs. none of the time	−0.71	0.14	−0.99	−0.42	< 0.001^a^
ATE	Some of the time vs. none of the time	−0.55	0.12	−0.79	−0.31	< 0.001^a^
ATE	Most of the time vs. none of the time	−0.61	0.14	−0.87	−0.34	< 0.001^a^
ATE	All of the time vs. none of the time	−0.40	0.12	−0.63	− 0.17	0.001^a^
B: Is there someone whom you can count on for emotional support (discuss problems or help you make a difficult decision)?						
Weighted average	None of the time	45.66	0.16	45.35	45.97	< 0.001^a^
Weighted average	A little of the time	45.45	0.09	45.28	45.62	< 0.001^a^
Weighted average	Some of the time	45.46	0.05	45.37	45.56	< 0.001^a^
Weighted average	Most of the time	45.37	0.07	45.24	45.50	< 0.001^a^
Weighted average	All of the time	45.75	0.03	45.70	45.81	< 0.001^a^
ATE	A little of the time vs. none of the time	−0.21	0.18	−0.57	0.14	0.238
ATE	Some of the time vs. none of the time	−0.20	0.17	−0.52	0.13	0.231
ATE	Most of the time vs. none of the time	−0.29	0.17	−0.63	0.04	0.089
ATE	All of the time vs. none of the time	0.09	0.16	−0.22	0.41	0.572
C: How often do you have a desired level of contact with someone whom you feel close to, trust, and can confide in?						
Weighted average	None of the time	45.45	0.25	44.97	45.94	< 0.001^a^
Weighted average	A little of the time	45.62	0.06	45.50	45.74	< 0.001^a^
Weighted average	Some of the time	45.53	0.03	45.46	45.60	< 0.001^a^
Weighted average	Most of the time	45.69	0.05	45.60	45.79	< 0.001^a^
Weighted average	All of the time	45.76	0.04	45.67	45.84	< 0.001^a^
ATE	A little of the time vs. none of the time	0.17	0.25	−0.33	0.67	0.509
ATE	Some of the time vs. none of the time	0.08	0.25	−0.41	0.57	0.753
ATE	Most of the time vs. none of the time	0.24	0.25	−0.25	0.73	0.340
ATE	All of the time vs. none of the time	0.30	0.25	−0.19	0.79	0.226
D: Number of friends or neighbors with whom you can casually share your concerns						
Weighted average	None	44.94	0.32	44.32	45.56	< 0.001^a^
Weighted average	One or two	45.54	0.04	45.47	45.61	< 0.001^a^
Weighted average	Three or more	45.70	0.03	45.65	45.76	< 0.001^a^
ATE	One or two vs. none	0.60	0.32	−0.02	1.23	0.060
ATE	Three or more vs. none	0.77	0.32	0.14	1.39	0.016^b^
Neighborhood social capital						
E: Neighbors trust each other						
Weighted average	Disagree	45.32	0.05	45.21	45.42	< 0.001^a^
Weighted average	Somewhat disagree	45.45	0.04	45.36	45.53	< 0.001^a^
Weighted average	Somewhat agree	45.81	0.03	45.75	45.88	< 0.001^a^
Weighted average	Agree	46.22	0.08	46.06	46.38	< 0.001^a^
ATE	Somewhat disagree vs. disagree	0.13	0.07	−0.01	0.27	0.065
ATE	Somewhat agree vs. disagree	0.50	0.06	0.37	0.62	< 0.001^a^
ATE	Agree vs. disagree	0.90	0.10	0.71	1.10	< 0.001^a^
F: Neighbors help each other.						
Weighted average	Disagree	45.33	0.06	45.22	45.44	< 0.001^a^
Weighted average	Somewhat disagree	45.45	0.04	45.36	45.54	< 0.001^a^
Weighted average	Somewhat agree	45.82	0.03	45.75	45.88	< 0.001^a^
Weighted average	Agree	46.04	0.08	45.89	46.20	< 0.001^a^
ATE	Somewhat disagree vs. disagree	0.12	0.07	−0.02	0.26	0.094
ATE	Somewhat agree vs. disagree	0.48	0.06	0.36	0.61	< 0.001^a^
ATE	Agree vs. disagree	0.71	0.10	0.52	0.90	< 0.001^a^

*ATE* average treatment effect, *PCS* Physical Component Summary

^a^*p* < 0.01, ^b^*p* < 0.05

The ATEs of the MCS scores are reported in Tables 5. Except for “a little of the time vs. none of the time” for questions A and B, the statistically significant ATEs within pairs of different levels of individual social capital were all positive (Table 5). Further, all ATEs relating to neighborhood social capital were positive and statistically significant (Table 5). These ATEs show that higher levels of social capital have a larger positive impact on mental health. We can identify a proportional relationship between the level of social capital and the scale of the ATE. In questions A, B, E, and F, the largest differences in the MCS score associated with the highest levels of social capital were approximately 1.0–1.6. Moreover, for questions C and D, the largest differences caused by social capital were approximately 3.6 and 4.4, respectively. The maximum effects of individual and neighborhood social capital were approximately 4.4 (question D) and 1.6 (question E), respectively. The results for the PCS and MCS scores imply that both individual and neighborhood social capital have some degree of positive impact on mental health, with this being particularly true for neighborhood social capital.

**Table 5:** ATEs for MCS scores across different indicators of social capital

Individual social capital		COEF	SE	95% CI		*p*
A: Is there someone available to you who shows you love and affection?						
Weighted average	None of the time	48.73	0.12	48.50	48.97	< 0.001^a^
Weighted average	A little of the time	47.28	0.09	47.10	47.46	< 0.001^a^
Weighted average	Some of the time	48.65	0.04	48.57	48.73	< 0.001^a^
Weighted average	Most of the time	48.80	0.07	48.66	48.95	< 0.001^a^
Weighted average	All of the time	49.75	0.03	49.69	49.81	< 0.001^a^
ATE	A little of the time vs. none of the time	−1.45	0.15	−1.75	−1.16	< 0.001^a^
ATE	Some of the time vs. none of the time	−0.08	0.13	−0.33	0.17	0.542
ATE	Most of the time vs. none of the time	0.07	0.14	−0.20	0.35	0.607
ATE	All of the time vs. none of the time	1.02	0.12	0.78	1.26	< 0.001^a^
B: Is there someone whom you can count on for emotional support (discuss problems or help you make a difficult decision)?						
Weighted average	None of the time	48.15	0.17	47.82	48.48	< 0.001^a^
Weighted average	A little of the time	46.87	0.09	46.69	47.06	< 0.001^a^
Weighted average	Some of the time	48.42	0.05	48.33	48.52	< 0.001^a^
Weighted average	Most of the time	48.60	0.06	48.47	48.72	< 0.001^a^
Weighted average	All of the time	49.78	0.03	49.72	49.83	< 0.001^a^
ATE	A little of the time vs. none of the time	−1.28	0.19	−1.65	−0.90	< 0.001^a^
ATE	Some of the time vs. none of the time	0.27	0.18	−0.07	0.62	0.120
ATE	Most of the time vs. none of the time	0.45	0.18	0.09	0.80	0.014^b^
ATE	All of the time vs. none of the time	1.63	0.17	1.29	1.96	< 0.001^a^
C: How often do you have a desired level of contact with someone whom you feel close to, trust, and can confide in?						
Weighted average	None of the time	46.26	0.27	45.74	46.78	< 0.001^a^
Weighted average	A little of the time	47.98	0.06	47.86	48.11	< 0.001^a^
Weighted average	Some of the time	48.96	0.03	48.89	49.02	< 0.001^a^
Weighted average	Most of the time	49.40	0.05	49.30	49.49	< 0.001^a^
Weighted average	All of the time	49.86	0.04	49.78	49.94	< 0.001^a^
ATE	A little of the time vs. none of the time	1.72	0.27	1.18	2.26	< 0.001^a^
ATE	Some of the time vs. none of the time	2.69	0.27	2.17	3.22	< 0.001^a^
ATE	Most of the time vs. none of the time	3.13	0.27	2.61	3.66	< 0.001^a^
ATE	All of the time vs. none of the time	3.60	0.27	3.07	4.12	< 0.001^a^
D: Number of friends or neighbors with whom you can casually share your concerns						
Weighted average	None	45.32	0.32	44.70	45.94	< 0.001^a^
Weighted average	One or two	48.39	0.04	48.32	48.46	< 0.001^a^
Weighted average	Three or more	49.67	0.03	49.62	49.73	< 0.001^a^
ATE	One or two vs. none	3.07	0.32	2.44	3.69	< 0.001^a^
ATE	Three or more vs. none	4.35	0.32	3.73	4.98	< 0.001^a^
Neighborhood social capital						
E: Neighbors trust each other.						
Weighted average	Disagree	48.33	0.06	48.22	48.44	< 0.001^a^
Weighted average	Somewhat disagree	48.84	0.04	48.75	48.92	< 0.001^a^
Weighted average	Somewhat agree	49.45	0.03	49.39	49.52	< 0.001^a^
Weighted average	Agree	49.92	0.08	49.76	50.08	< 0.001^a^
ATE	Somewhat disagree vs. disagree	0.51	0.07	0.37	0.64	< 0.001^a^
ATE	Somewhat agree vs. disagree	1.12	0.06	1.00	1.25	< 0.001^a^
ATE	Agree vs. disagree	1.59	0.10	1.40	1.79	< 0.001^a^
F: Neighbors help each other.						
Weighted average	Disagree	48.41	0.06	48.29	48.52	< 0.001^a^
Weighted average	Somewhat disagree	48.84	0.04	48.75	48.92	< 0.001^a^
Weighted average	Somewhat agree	49.39	0.03	49.33	49.45	< 0.001^a^
Weighted average	Agree	49.82	0.08	49.67	49.96	< 0.001^a^
ATE	Somewhat disagree vs. disagree	0.43	0.07	0.29	0.57	< 0.001^a^
ATE	Somewhat agree vs. disagree	0.99	0.06	0.86	1.11	< 0.001^a^
ATE	Agree vs. disagree	1.41	0.09	1.22	1.59	< 0.001^a^

*ATE* average treatment effect, *MCS* Mental Component Summary

^a^*p* < 0.01, ^b^*p* < 0.05

## Discussion

Our research contributes to existing literature by identifying the positive impact social capital has on the mental health of pregnant women. Furthermore, by using nationwide survey data collected across Japan, the generalizability of our findings relating to social capital and health during pregnancy is high; in comparison, a previous work that used scores obtained from the SF-12 [4] examined fewer than 1000 participants.

Our results indicate that, for pregnant women, a lack of social ties is associated with worse health; this was especially notable in regard to mental health during pregnancy. This finding, showing that the mental health of pregnant women is improved by social capital, can have important implications for the future practice, and also suggests that Japanese people should endeavor to construct appropriate social ties.

It is also important to explain the relevance of this study in terms of comparing its design with that of previous related research. Studies that have measured the effect of social capital on health can be categorized into four groups in terms of the data type examined: (1) individual social capital and health outcomes, (2) individual social capital and group-level health outcomes, (3) group-level social capital and individual health outcomes, and (4) group-level social capital and health outcomes [1]. Our study can be categorized into designs 1 and 3. PCS and MCS scores, individual-level social capital, and group-level social capital were measured through the participants’ responses. The question items relating to social capital required participants to provide information regarding their communication network at the individual level and to evaluate their degree of trust in and support received from their neighbors. This evaluation indicates their neighbors’ group attributes and is regarded as a collective factor. The variables of individual and neighborhood social capital indicate the degree of individual network resources available to participants and the participants’ social cohesion, respectively.

This study differs from previous studies that have analyzed the influence of social capital using JECS data [9, 10]. One such study, which used the Kessler 6-Item Psychological Distress Scale (K6) as an outcome measure, did not consider the neighborhood social capital data obtained through questions E and F [9]. Another study considered gestational diabetes mellitus as an outcome; while this research used the question items we applied in our analysis, the researchers also examined responses to question items concerning the degree of regional public safety, mutual trust, and mutual assistance through principal component analysis [10]. The JECS question items for mutual trust and assistance are: “Would you say that most people can be trusted?” and “Would you say that most of the time people try to be helpful, or that they are mostly thinking of themselves?” This measure of generalized trust is debatable, because the question items do not specify a reference area for the respondent [41]. Further, questions that ask about generalized trust may cause respondents to report certain perceptions that are unrelated to their life within their communities [41]. Therefore, such question items are gradually being removed in favor of items that refer to familiar or personal trust [41]. The question items in the JECS survey regarding regional public safety and mutual assistance also present similar problems, as they do not clearly specify a reference area. Our study regarded both individual networking and social cohesion as social capital to be investigated, and our analysis purposely did not include question items concerning regional public safety and generalized feelings.

A limitation to our analysis is that we did not obtain detailed information related to social capital, such as friends’ and neighbors’ characteristics. Network analysis of social capital, using the “position generator” and “resource generator” measurement instruments, could identify the effectiveness of individual network members [42]. However, identification of substantial functions among network members is difficult in analysis of nationwide survey data sourced from a large number of question items.

## Conclusions

We used JECS data to analyze the impact of social capital on the health of pregnant women in Japan. We calculated ATEs of social capital on the PCS and MCS scores of the SF-8 using the IPW estimator. We consequently found that social capital has a degree of positive influence on MCS score. This result implies that enhancing social capital would contribute to improving women’s mental health during pregnancy.

## Supplementary information

**Additional file 1: Supplementary Table 1.** Items that assess social capital.

**Additional file 2: Supplementary Table 2-1.** Balance check using standardized differences for individual social capital.

**Additional file 3: Supplementary Table 2–2.** Balance check using standardized differences for individual social capital.

**Additional file 4: Supplementary Table 2–3.** Balance check using standardized differences for neighborhood social capital.

## Data Availability

The data used to derive our conclusions are unsuitable for public deposition owing to ethical restrictions and the specific legal framework in Japan. Specifically, it is prohibited by the Act on the Protection of Personal Information (Act No. 57 of 30 May 2003, amended 9 September 2015) to publicly deposit data containing personal information. The Ethical Guidelines for Epidemiological Research enforced by the Japan Ministry of Education, Culture, Sports, Science and Technology and the Ministry of Health, Labour and Welfare also restrict the open sharing of epidemiologic data. All inquiries regarding access to the data should be sent to jecs-en@nies.go.jp. The person responsible for handling inquiries sent to this e-mail address is Dr. Shoji F. Nakayama, JECS Program Office, National Institute for Environmental Studies.

## Abbreviations

ATE: Average treatment effect
CI: Confidence interval
COEF: Coefficient
IPW: Inverse probability weighting
JECS: Japan Environment and Children’s Study
K6: Kessler 6-Item Psychological Distress Scale
MCS: Mental Component Summary
OHRQoL: Oral health-related quality of life
PCS: Physical Component Summary
PHDCN: Project on Human Development in Chicago Neighborhoods
SD: Standard deviation
SE: Standard error
SF-8: 8-Item Short-Form Health Survey
SF-12: 12-Item Short-Form Health Survey
SF-36: 36-Item Short-Form Health Survey
SSQ: Social Support Questionnaire
