# Supplementary material for: Impact of individual and neighborhood social capital on the physical and mental health of pregnant women: the Japan Environment and Children’s Study (JECS)
Source: BMC Pregnancy Childbirth. 2020 Aug 6;20:450. doi: 10.1186/s12884-020-03131-3 (PMC7409696; doi:10.1186/s12884-020-03131-3)
Supplement: Supplementary file 1 — Additional file 1: Supplementary Table 1. Items that assess social capital. [file 12884_2020_3131_MOESM1_ESM.docx]

**Supplementary Table 1.** Items that assess social capital

| Individual social capital |  |  |  |  |  |  |  |
| --- | --- | --- | --- | --- | --- | --- | --- |
| A: Is there someone available to you who shows you love and affection? | | | | |  |  |  |
| (1) None of the time, (2) A little of the time, (3) Some of the time, (4) Most of the time, (5) All of the time | | | | | | |  |
| B: Is there someone whom you can count on for emotional support  (discuss problems or help you make a difficult decision)? | | | | | | |  |
| (1) None of the time, (2) A little of the time, (3) Some of the time, (4) Most of the time, (5) All of the time | | | | | | |  |
| C: How often do you have a desired level of contact with someone whom you feel close to, trust, and can confide in? | | | | | | |  |
| (1) None of the time, (2) A little of the time, (3) Some of the time, (4) Most of the time, (5) All of the time | | | | | | |  |
| D: Number of friends or neighbors with whom you can casually share your concerns | | | | |  |  |  |
| (1) None, (2) One or two, (3) Three or more | | |  |  |  |  |  |
| Neighborhood social capital | |  |  |  |  |  |  |
| E: Neighbors trust each other. | |  |  |  |  |  |  |
| (1) Agree, (2) Somewhat agree, (3) Somewhat disagree, (4) Disagree | | | |  |  |  |  |
| F: Neighbors help each other. | |  |  |  |  |  |  |
| (1) Agree, (2) Somewhat agree, (3) Somewhat disagree, (4) Disagree | | | |  |  |  |  |
