# Supplementary material for: Impact of individual and neighborhood social capital on the physical and mental health of pregnant women: the Japan Environment and Children’s Study (JECS)
Source: BMC Pregnancy Childbirth. 2020 Aug 6;20:450. doi: 10.1186/s12884-020-03131-3 (PMC7409696; doi:10.1186/s12884-020-03131-3)
Supplement: Supplementary file 2 — Additional file 2: Supplementary Table 2-1. Balance check using standardized differences for individual social capital. [file 12884_2020_3131_MOESM2_ESM.docx]

**Supplementary Table 2-1.** Balance check using standardized differences for individual social capital

| Individual social capital | A: Is there someone available to you | | B: Is there someone whom you can count on for | | C: How often do you have a desired | |
| --- | --- | --- | --- | --- | --- | --- |
|  | who shows you love and affection? | | emotional support  (discuss problems | | level of contact with someone whom you feel | |
|  |  |  | or help you make a | | close to, trust, and can | |
|  |  |  | difficult decision)? | | confide in? | |
| Characteristics | Raw | Weighted | Raw | Weighted | Raw | Weighted |
| A little of the time |  |  |  |  |  |  |
| Age | -0.05 | 0.01 | -0.08 | 0.01 | 0.13 | 0.01 |
| Married or in a common-law relationship | -0.13 | -0.01 | -0.11 | -0.01 | 0.07 | 0.00 |
| One or more children | -0.09 | 0.01 | -0.03 | 0.01 | -0.07 | -0.01 |
| Self-reported history of disease | 0.05 | -0.01 | 0.01 | 0.01 | 0.05 | 0.02 |
| Obstetric complications | 0.04 | 0.00 | -0.03 | 0.00 | 0.02 | -0.01 |
| Experience of any stressful events | 0.21 | 0.00 | 0.17 | 0.01 | 0.01 | 0.01 |
| Labor force participation | 0.04 | -0.01 | 0.02 | 0.00 | 0.13 | 0.00 |
| Mother's academic history |  |  |  |  |  |  |
| Technical college or vocational school | -0.01 | 0.00 | -0.04 | -0.01 | 0.06 | 0.01 |
| College, university, or graduate school | -0.14 | 0.00 | -0.16 | 0.00 | 0.27 | -0.01 |
| Father's academic history |  |  |  |  |  |  |
| Technical college or vocational school | 0.01 | -0.01 | 0.01 | -0.01 | 0.06 | 0.04 |
| College, university, or graduate school | -0.10 | 0.01 | -0.14 | 0.01 | 0.20 | -0.04 |
| Household income (million JPY/year) |  |  |  |  |  |  |
| 2–4 | 0.04 | 0.00 | 0.11 | 0.00 | -0.09 | 0.00 |
| 4–6 | -0.04 | 0.00 | -0.04 | 0.01 | 0.06 | 0.01 |
| 6–8 | -0.05 | 0.00 | -0.10 | 0.00 | 0.15 | 0.01 |
| 8–10 | -0.06 | 0.01 | -0.09 | 0.00 | 0.10 | -0.02 |
| ≥ 10 | -0.02 | 0.00 | -0.03 | -0.01 | 0.07 | -0.03 |
| Some of the time |  |  |  |  |  |  |
| Age | 0.07 | 0.00 | 0.01 | 0.00 | 0.18 | 0.01 |
| Married or in a common-law relationship | 0.00 | -0.01 | 0.00 | -0.01 | 0.11 | 0.00 |
| One or more children | -0.12 | 0.01 | -0.04 | 0.00 | -0.12 | -0.02 |
| Self-reported history of disease | 0.08 | 0.00 | -0.03 | 0.00 | 0.06 | 0.02 |
| Obstetric complications | 0.05 | 0.00 | -0.01 | 0.00 | 0.03 | 0.00 |
| Experience of any stressful events | 0.10 | 0.00 | -0.01 | 0.01 | -0.11 | 0.01 |
| Labor force participation | 0.07 | -0.01 | 0.06 | -0.01 | 0.11 | -0.01 |
| Mother's academic history |  |  |  |  |  |  |
| Technical college or vocational school | 0.05 | 0.01 | 0.01 | 0.00 | 0.06 | 0.01 |
| College, university, or graduate school | 0.07 | -0.01 | -0.01 | -0.01 | 0.40 | -0.01 |
| Father's academic history |  |  |  |  |  |  |
| Technical college or vocational school | 0.08 | 0.00 | 0.06 | 0.00 | 0.08 | 0.04 |
| College, university, or graduate school | 0.03 | 0.00 | -0.05 | 0.00 | 0.28 | -0.04 |
| Household income (million JPY/year) |  |  |  |  |  |  |
| 2–4 | -0.02 | 0.00 | 0.03 | 0.00 | -0.15 | 0.00 |
| 4–6 | 0.02 | 0.00 | 0.04 | 0.00 | 0.10 | 0.01 |
| 6–8 | 0.04 | 0.00 | 0.01 | 0.00 | 0.20 | 0.01 |
| 8–10 | -0.01 | 0.00 | -0.06 | 0.00 | 0.15 | -0.02 |
| ≥ 10 | 0.03 | 0.00 | 0.01 | -0.01 | 0.11 | -0.03 |
| Most of the time |  |  |  |  |  |  |
| Age | 0.11 | 0.00 | 0.03 | 0.00 | 0.13 | 0.00 |
| Married or in a common-law relationship | 0.01 | -0.01 | 0.00 | -0.01 | 0.14 | 0.00 |
| One or more children | -0.15 | 0.00 | -0.06 | 0.00 | -0.16 | -0.02 |
| Self-reported history of disease | 0.10 | 0.00 | 0.02 | 0.00 | 0.07 | 0.02 |
| Obstetric complications | 0.04 | 0.00 | -0.01 | 0.00 | 0.03 | 0.00 |
| Experience of any stressful events | 0.07 | 0.00 | 0.01 | 0.01 | -0.14 | 0.01 |
| Labor force participation | 0.10 | -0.01 | 0.07 | 0.00 | 0.08 | -0.01 |
| Mother's academic history |  |  |  |  |  |  |
| Technical college or vocational school | 0.04 | 0.00 | 0.01 | 0.00 | 0.06 | 0.01 |
| College, university, or graduate school | 0.20 | -0.01 | 0.12 | -0.01 | 0.48 | -0.01 |
| Father's academic history |  |  |  |  |  |  |
| Technical college or vocational school | 0.08 | 0.00 | 0.05 | 0.00 | 0.09 | 0.04 |
| College, university, or graduate school | 0.11 | 0.00 | 0.04 | 0.00 | 0.34 | -0.04 |
| Household income (million JPY/year) |  |  |  |  |  |  |
| 2–4 | -0.15 | 0.00 | -0.06 | 0.00 | -0.18 | 0.00 |
| 4–6 | 0.09 | 0.00 | 0.08 | 0.00 | 0.12 | 0.01 |
| 6–8 | 0.11 | 0.00 | 0.05 | 0.00 | 0.21 | 0.01 |
| 8–10 | 0.05 | 0.00 | 0.01 | 0.00 | 0.16 | -0.02 |
| ≥ 10 | 0.07 | 0.00 | 0.04 | -0.01 | 0.13 | -0.03 |
| All of the time |  |  |  |  |  |  |
| Age | 0.15 | 0.00 | 0.02 | 0.00 | 0.16 | 0.01 |
| Married or in a common-law relationship | 0.07 | -0.01 | 0.07 | -0.01 | 0.13 | 0.00 |
| One or more children | -0.18 | 0.01 | -0.09 | 0.00 | -0.12 | -0.02 |
| Self-reported history of disease | 0.13 | 0.00 | 0.01 | 0.00 | 0.07 | 0.02 |
| Obstetric complications | 0.02 | 0.00 | -0.03 | 0.00 | 0.03 | 0.00 |
| Experience of any stressful events | 0.04 | 0.00 | -0.09 | 0.01 | -0.18 | 0.01 |
| Labor force participation | 0.04 | -0.01 | 0.03 | 0.00 | 0.07 | 0.00 |
| Mother's academic history |  |  |  |  |  |  |
| Technical college or vocational school | 0.04 | 0.01 | 0.01 | 0.00 | 0.03 | 0.01 |
| College, university, or graduate school | 0.30 | -0.01 | 0.17 | -0.01 | 0.56 | -0.01 |
| Father's academic history |  |  |  |  |  |  |
| Technical college or vocational school | 0.07 | 0.00 | 0.07 | 0.00 | 0.07 | 0.04 |
| College, university, or graduate school | 0.21 | 0.00 | 0.10 | 0.00 | 0.40 | -0.04 |
| Household income (million JPY/year) |  |  |  |  |  |  |
| 2–4 | -0.17 | 0.00 | -0.08 | 0.00 | -0.22 | 0.00 |
| 4–6 | 0.06 | 0.00 | 0.07 | 0.00 | 0.11 | 0.01 |
| 6–8 | 0.13 | 0.00 | 0.06 | 0.00 | 0.23 | 0.01 |
| 8–10 | 0.09 | 0.00 | 0.03 | 0.00 | 0.19 | -0.02 |
| ≥ 10 | 0.12 | 0.00 | 0.08 | -0.01 | 0.16 | -0.03 |
