# Supplementary material for: Impact of individual and neighborhood social capital on the physical and mental health of pregnant women: the Japan Environment and Children’s Study (JECS)
Source: BMC Pregnancy Childbirth. 2020 Aug 6;20:450. doi: 10.1186/s12884-020-03131-3 (PMC7409696; doi:10.1186/s12884-020-03131-3)
Supplement: Supplementary file 3 — Additional file 3: Supplementary Table 2–2. Balance check using standardized differences for individual social capital. [file 12884_2020_3131_MOESM3_ESM.docx]

**Supplementary Table 2-2.** Balance check using standardized differences for individual social capital

| Individual social capital | D: Number of friends or neighbors with | |
| --- | --- | --- |
|  | whom you can casually share your | |
|  | concerns |  |
| Characteristics | Raw | Weighted |
| One or two |  |  |
| Age | 0.07 | -0.02 |
| Married or in a common-law relationship | 0.18 | 0.00 |
| One or more children | -0.02 | -0.06 |
| Self-reported history of disease | -0.07 | -0.02 |
| Obstetric complications | 0.02 | 0.03 |
| Experience of any stressful events | -0.27 | -0.01 |
| Labor force participation | 0.12 | 0.03 |
| Mother's academic history |  |  |
| Technical college or vocational school | 0.06 | 0.00 |
| College, university, or graduate school | 0.28 | 0.02 |
| Father's academic history |  |  |
| Technical college or vocational school | 0.09 | 0.01 |
| College, university, or graduate school | 0.17 | -0.01 |
| Household income (million JPY/year) |  |  |
| 2–4 | -0.09 | 0.01 |
| 4–6 | 0.12 | 0.00 |
| 6–8 | 0.15 | -0.01 |
| 8–10 | 0.11 | 0.02 |
| ≥ 10 | 0.07 | -0.02 |
| Three or more |  |  |
| Age | 0.07 | -0.02 |
| Married or in a common-law relationship | 0.24 | 0.00 |
| One or more children | -0.07 | -0.06 |
| Self-reported history of disease | -0.06 | -0.02 |
| Obstetric complications | 0.01 | 0.03 |
| Experience of any stressful events | -0.37 | -0.01 |
| Labor force participation | 0.13 | 0.03 |
| Mother's academic history |  |  |
| Technical college or vocational school | 0.08 | 0.00 |
| College, university, or graduate school | 0.41 | 0.02 |
| Father's academic history |  |  |
| Technical college or vocational school | 0.12 | 0.01 |
| College, university, or graduate school | 0.24 | -0.01 |
| Household income (million JPY/year) |  |  |
| 2–4 | -0.16 | 0.01 |
| 4–6 | 0.15 | 0.00 |
| 6–8 | 0.22 | -0.01 |
| 8–10 | 0.16 | 0.02 |
| ≥ 10 | 0.10 | -0.02 |
