# Supplementary material for: Impact of individual and neighborhood social capital on the physical and mental health of pregnant women: the Japan Environment and Children’s Study (JECS)
Source: BMC Pregnancy Childbirth. 2020 Aug 6;20:450. doi: 10.1186/s12884-020-03131-3 (PMC7409696; doi:10.1186/s12884-020-03131-3)
Supplement: Supplementary file 4 — Additional file 4: Supplementary Table 2–3. Balance check using standardized differences for neighborhood social capital. [file 12884_2020_3131_MOESM4_ESM.docx]

**Supplementary Table 2-3.** Balance check using standardized differences for neighborhood social capital

| Neighborhood social capital | E: Neighbors trust each other. | | F: Neighbors help each other. | |
| --- | --- | --- | --- | --- |
| Characteristics | Raw | Weighted | Raw | Weighted |
| Somewhat disagree |  |  |  |  |
| Age | 0.19 | 0.02 | 0.22 | 0.02 |
| Married or in a common-law relationship | 0.08 | 0.01 | 0.08 | 0.01 |
| One or more children | 0.23 | 0.02 | 0.23 | 0.02 |
| Self-reported history of disease | 0.00 | 0.01 | 0.02 | 0.01 |
| Obstetric complications | -0.02 | 0.00 | -0.02 | 0.00 |
| Experience of any stressful events | -0.04 | 0.00 | -0.04 | 0.00 |
| Labor force participation | -0.07 | -0.01 | -0.07 | -0.01 |
| Mother's academic history |  |  |  |  |
| Technical college or vocational school | -0.01 | 0.00 | -0.02 | 0.00 |
| College, university, or graduate school | 0.08 | 0.01 | 0.12 | 0.02 |
| Father's academic history |  |  |  |  |
| Technical college or vocational school | 0.01 | 0.00 | 0.00 | 0.00 |
| College, university, or graduate school | 0.03 | 0.01 | 0.06 | 0.01 |
| Household income (million JPY/year) |  |  |  |  |
| 2–4 | -0.03 | 0.00 | -0.04 | 0.00 |
| 4–6 | 0.03 | 0.01 | 0.02 | 0.01 |
| 6–8 | 0.03 | 0.00 | 0.04 | 0.00 |
| 8–10 | 0.02 | 0.00 | 0.03 | 0.00 |
| ≥ 10 | 0.02 | 0.00 | 0.04 | 0.00 |
| Somewhat agree |  |  |  |  |
| Age | 0.35 | 0.01 | 0.36 | 0.01 |
| Married or in a common-law relationship | 0.14 | 0.01 | 0.13 | 0.01 |
| One or more children | 0.47 | 0.01 | 0.50 | 0.01 |
| Self-reported history of disease | 0.00 | 0.01 | 0.00 | 0.01 |
| Obstetric complications | -0.04 | 0.00 | -0.04 | 0.00 |
| Experience of any stressful events | -0.09 | 0.00 | -0.07 | 0.00 |
| Labor force participation | -0.17 | -0.01 | -0.17 | -0.01 |
| Mother's academic history |  |  |  |  |
| Technical college or vocational school | -0.03 | 0.00 | -0.02 | 0.00 |
| College, university, or graduate school | 0.19 | 0.01 | 0.15 | 0.02 |
| Father's academic history |  |  |  |  |
| Technical college or vocational school | 0.02 | 0.00 | 0.01 | 0.00 |
| College, university, or graduate school | 0.09 | 0.01 | 0.06 | 0.01 |
| Household income (million JPY/year) |  |  |  |  |
| 2–4 | -0.09 | -0.01 | -0.08 | -0.01 |
| 4–6 | 0.06 | 0.01 | 0.06 | 0.01 |
| 6–8 | 0.06 | 0.00 | 0.04 | 0.00 |
| 8–10 | 0.03 | 0.00 | 0.03 | 0.00 |
| ≥ 10 | 0.08 | 0.00 | 0.08 | 0.00 |
| Agree |  |  |  |  |
| Age | 0.41 | -0.01 | 0.36 | 0.00 |
| Married or in a common-law relationship | 0.16 | -0.01 | 0.14 | 0.00 |
| One or more children | 0.65 | 0.00 | 0.65 | 0.01 |
| Self-reported history of disease | -0.07 | 0.00 | -0.06 | 0.00 |
| Obstetric complications | -0.06 | 0.01 | -0.04 | 0.01 |
| Experience of any stressful events | -0.18 | 0.01 | -0.13 | 0.01 |
| Labor force participation | -0.28 | 0.00 | -0.28 | 0.00 |
| Mother's academic history |  |  |  |  |
| Technical college or vocational school | -0.04 | 0.00 | -0.03 | 0.00 |
| College, university, or graduate school | 0.22 | -0.01 | 0.15 | 0.00 |
| Father's academic history |  |  |  |  |
| Technical college or vocational school | 0.00 | 0.00 | 0.01 | 0.00 |
| College, university, or graduate school | 0.14 | -0.01 | 0.06 | -0.01 |
| Household income (million JPY/year) |  |  |  |  |
| 2–4 | -0.11 | 0.01 | -0.07 | 0.01 |
| 4–6 | 0.07 | -0.01 | 0.05 | -0.01 |
| 6–8 | 0.03 | -0.01 | 0.01 | 0.00 |
| 8–10 | 0.05 | 0.00 | 0.04 | 0.00 |
| ≥ 10 | 0.11 | 0.00 | 0.10 | 0.00 |
